# Supplementary material for: Disparities in Smokefree and Vapefree Home Rules and Smokefree Policy Attitudes Based on Housing Type and Cigarette Smoking Status, United States, 2019
Source: Int J Environ Res Public Health. 2023 Jul 13;20(14):6356. doi: 10.3390/ijerph20146356 (PMC10379655; doi:10.3390/ijerph20146356)
Supplement: Supplementary file 1 [file ijerph-20-06356-s001.zip › ijerph-2339340-supplementary.pdf]

Supplementary Table S1. Participant Sociodemographic, Occupation, and Housing Tenure Characteristics Among Multi-Unit and Single-Unit Housing Residents by Smoking Status, 2019

	MULTI-UNIT HOUSING					SINGLE-UNIT HOUSING				
**Variable**	**N= 59,910,550**	**Total (n=9,662; 24%)**	**Current smoking (n=1,151; 10.8%)**	**Former smoking (n=1,850; 15.6%)**	**Never smoking (n=6,661; 73.6%)**	**N= 162,349,276**	**Total (n=30,634; 76%)**	**Current smoking (n=3,484; 10.7%)**	**Former smoking (n=6,797; 19.3%)**	**Never smoking (n=20,353; 70.0%)**
	N	n	n (%)	n (%)	n (%)	N	n	n (%)	n (%)	n (%)
**SEX**										
Male	28,299,086	4,288	567 (55.0)	949 (55.4)	2,772 (44.6)	78,694,646	14,139	1,772 (54.4)	3,739 (56.7)	8,628 (45.3)
Female	31,611,464	5,374	584 (45.0)	901 (45.6)	3,889 (55.4)	83,654,631	16,495	1,712 (45.6)	3,058 (43.3)	11,725 (54.7)
**AGE GROUP**										
18–24	9,062,985	822	63 (9.3)	46 (4.1)	713 (18.3)	15,342,429	1,158	98 (5.0)	60 (1.9)	1,000 (12.2)
25–34	14,876,436	2,196	203 (19.1)	227 (15.0)	1,766 (27.8)	23,971,909	3,924	501 (17.8)	499 (8.7)	2,924 (16.0)
35–44	9,448,460	1,536	198 (17.1)	250 (15.5)	1,088 (15.6)	26,684,238	4,972	639 (18.4)	835 (13.3)	3,498 (17.0)
45–54	8,104,164	1,281	197 (18.3)	203 (12.1)	881 (13.1)	28,315,298	4,827	669 (20.3)	836 (15.4)	3,322 (17.6)
55+	18,418,504	3,827	490 (36.2)	1,124 (53.4)	2,213 (25.2)	68,035,402	15,753	1,577 (38.6)	4,567 (60.6)	9,609 (37.3)
**RACE/ETHNICITY**										
Hispanic	13,624,968	1,574	106 (13.6)	192 (15.4)	1,276 (25.6)	22,474,702	2,748	188 (8.4)	344 (8.0)	2,216 (16.3)
White, non-Hispanic	29,233,229	5,745	728 (54.2)	1,357 (65.3)	3,660 (44.5)	112,857,421	23,852	2,830 (75.3)	5,872 (81.7)	15,150 (65.3)
Black, non-Hispanic	10,227,364	1,398	225 (23.4)	183 (11.1)	990 (17.4)	14,860,913	2,323	299 (11.2)	320 (5.5)	1,704 (9.9)
Other	6,824,989	945	92 (8.8)	118 (8.3)	735 (12.4)	12,156,241	1,711	167 (5.2)	261 (4.8)	1,283 (8.6)
**REGION**										

	MULTI-UNIT HOUSING					SINGLE-UNIT HOUSING				
**Variable**	**N= 59,910,550**	**Total (n=9,662; 24%)**	**Current smoking (n=1,151; 10.8%)**	**Former smoking (n=1,850; 15.6%)**	**Never smoking (n=6,661; 73.6%)**	**N= 162,349,276**	**Total (n=30,634; 76%)**	**Current smoking (n=3,484; 10.7%)**	**Former smoking (n=6,797; 19.3%)**	**Never smoking (n=20,353; 70.0%)**
Northeast	14,827,997	2,159	237 (22.5)	449 (25.6)	1,473 (24.9)	24,137,297	4,208	409 (13.6)	1,124 (17.0)	2,675 (14.5)
Midwest	10,449,451	1,788	258 (23.7)	399 (20.7)	1,131 (15.8)	36,429,375	6,656	832 (25.5)	1,576 (25.0)	4,248 (21.3)
South	18,442,502	2,959	368 (31.3)	504 (27.5)	2,087 (31.4)	65,160,922	11,808	1,507 (44.4)	2,402 (36.5)	7,899 (40.5)
West	16,190,600	2,756	288 (22.5)	498 (26.2)	1,970 (27.9)	36,621,683	7,962	736 (16.5)	1,695 (21.6)	5,531 (23.8)
**METROPOLITAN STATUS**										
Metropolitan	56,857,572	8,717	984 (91.3)	1,631 (92.9)	6,102 (95.9)	137,281,468	23,255	2,376 (77.5)	5,104 (83.2)	15,775 (86.0)
Non-metropolitan	3,052,978	945	167 (8.7)	219 (7.1)	559 (4.1)	25,067,809	7,379	1,108 (22.5)	1,693 (16.8)	4,578 (14.0)
**ANNUAL HOUSEHOLD INCOME**										
<$20,000	12,536,686	2,108	416 (34.0)	419 (21.4)	1,273 (18.9)	16,160,231	3,375	707 (18.9)	746 (9.9)	1,922 (8.6)
$20,000–$39,999	15,405,301	2,435	329 (28.2)	461 (24.2)	1,645 (25.7)	29,384,622	5,911	898 (24.3)	1,477 (20.3)	3,536 (16.6)
$40,000–$74,999	15,669,212	2,463	257 (23.1)	489 (27.2)	1,717 (26.4)	42,356,634	8,386	1,044 (30.1)	2,004 (28.8)	5,338 (24.7)
$75,000+	16,299,351	2,656	149 (14.7)	481 (27.3)	2,026 (29.0)	74,447,789	12,962	835 (26.7)	2,570 (41.0)	9,557 (50.1)
**EDUCATIONAL ATTAINMENT**										
Less than high school	6,932,963	1,020	186 (16.2)	183 (10.7)	651 (11.1)	15,109,773	2,413	478 (14.1)	523 (7.8)	1,412 (9.0)
High school	14,764,316	2,390	438 (38.6)	488 (25.1)	1,464 (22.5)	42,264,346	8,193	1,397 (40.4)	1,997 (28.7)	4,799 (23.1)

	MULTI-UNIT HOUSING					SINGLE-UNIT HOUSING				
**Variable**	**N= 59,910,550**	**Total (n=9,662; 24%)**	**Current smoking (n=1,151; 10.8%)**	**Former smoking (n=1,850; 15.6%)**	**Never smoking (n=6,661; 73.6%)**	**N= 162,349,276**	**Total (n=30,634; 76%)**	**Current smoking (n=3,484; 10.7%)**	**Former smoking (n=6,797; 19.3%)**	**Never smoking (n=20,353; 70.0%)**
Some college, no degree	10,671,223	1,657	219 (18.5)	390 (21.3)	1,048 (17.0)	29,669,450	5,407	712 (20.4)	1,371 (20.3)	3,324 (17.4)
Associate degree	5,683,588	925	114 (10.0)	203 (10.4)	608 (9.2)	17,192,056	3,451	398 (10.7)	816 (11.7)	2,237 (10.3)
College degree	21,858,460	3,670	194 (16.8)	586 (32.6)	2,890 (40.2)	58,113,652	11,170	499 (14.4)	2,090 (31.6)	8,581 (40.2)
**EMPLOYMENT STATUS**										
Working/Employed-at work/With job, not at work/Employed absent	38,202,922	5,803	604 (55.5)	891 (52.7)	4,308 (67.3)	100,726,370	17,752	1,956 (59.7)	3,273 (52.8)	12,523 (65.0)
Unemployed-on layoff/Unemployed-looking for work	2,126,112	296	50 (4.8)	56 (3.3)	190 (3.4)	3,723,390	593	129 (3.9)	111 (1.8)	353 (2.2)
Not in labor force (Retired, Disabled, Other)	19,581,516	3,563	497 (39.7)	903 (44.0)	2,163 (29.3)	57,899,516	12,289	1,399 (36.4)	3,413 (45.4)	7,477 (32.9)
**OCCUPATIONAL CATEGORY**										
White collar	26,185,243	4,099	330 (29.6)	635 (37.9)	3,134 (47.0)	68,581,450	12,465	985 (29.0)	2,249 (35.9)	9,231 (46.0)
Service	7,197,576	1,048	154 (13.2)	142 (7.8)	752 (12.7)	15,111,113	2,465	424 (12.6)	439 (7.0)	1,602 (9.4)
Blue collar	6,752,849	942	171 (17.6)	178 (10.9)	593 (10.4)	20,625,118	3,376	675 (22.0)	701 (11.8)	2,000 (11.6)
Other/Not in labor force	19,774,882	3,573	496 (39.7)	895 (43.5)	2,182 (29.8)	58,031,595	12,328	1,400 (36.4)	3,408 (45.4)	7,520 (33.0)

	MULTI-UNIT HOUSING					SINGLE-UNIT HOUSING				
**Variable**	**N= 59,910,550**	**Total (n=9,662; 24%)**	**Current smoking (n=1,151; 10.8%)**	**Former smoking (n=1,850; 15.6%)**	**Never smoking (n=6,661; 73.6%)**	**N= 162,349,276**	**Total (n=30,634; 76%)**	**Current smoking (n=3,484; 10.7%)**	**Former smoking (n=6,797; 19.3%)**	**Never smoking (n=20,353; 70.0%)**
**HOUSING TENURE**										
Public	5,182,886	905	188 (15.5)	194 (9.5)	523 (7.5)	1,082,507	198	44 (1.2)	42 (0.6)	112 (0.6)
Private owned	15,278,762	2,659	203 (17.6)	638 (33.2)	1,818 (25.0)	131,991,591	25,684	2,522 (69.2)	5,926 (85.5)	17,236 (82.0)
Private rented*	39,448,902	6,098	760 (66.9)	1,018 (57.4)	4,320 (67.5)	29,275,178	4,752	918 (29.6)	829 (13.9)	3,005 (17.4)

Notes:

1. Multi-unit housing is defined as two or more housing units that share a wall or ceiling. Single-unit housing refers to independent housing units, such as single-family homes, mobile homes, and other housing units that do not share a wall or ceiling.
2. All percentages are weighted.
3. P-values for all chi-squared tests comparing each characteristic by housing type and smoking status, were statistically significant at $p<0.0001$.
4. Smoking status was missing for 53 observations with multi-unit housing and 153 among observations with single-unit housing. Metropolitan status was missing for 60 with multi-unit housing and 394 with single-unit housing. Weighted percentages were calculated without these missing observations and were excluded from the analysis using a subdomain statement.

*Rent includes no cash rent or rent without payment.

Supplementary Table S2. Association of Smoking Status with Presence of Smokefree Rules (2a) and Vapefree Rules (2b) Inside Residents Homes, by Housing Type, 2019

	Table 2a<br>aOR Presence of Smokefree Rules—CIGARETTES (Complete vs Partial/None) among Multi- and Single-Unit Housing Residents				Table 2b<br>aOR Presence of Vapefree Rules—E-CIGARETTES (Complete vs Partial/None) among Multi- and Single-Unit Housing Residents			
**Variable**	**Unweighted N=39,624<br>Weighted N=218,409,352<br>aOR (95% CI)**				**Unweighted N=39,444<br>Weighted N=217,452,139<br>aOR (95% CI)**			
	**MUH Complete Rule**	**MUH Partial/No Rule**	**SUH Complete Rule**	**SUH Partial/No Rule (ref)**	**MUH Complete Rule**	**MUH Partial/No Rule**	**SUH Complete Rule**	**SUH Partial/No Rule (ref)**
**SEX**								
Male	0.95 (0.76-1.20)	1.08 (0.76-1.54)	0.94 (0.77-1.15)	1	0.91 (0.73-1.14)	1.11 (0.77-1.59)	0.91 (0.76-1.09)	1
Female	1				1			
**AGE GROUP**								
18–24	1.03 (0.59-1.81)	1.30 (0.56-3.01)	1.09 (0.62-1.92)	1	**0.44 (0.27-0.74)**	1.45 (0.70-3.01)	**0.50 (0.31-0.79)**	1
25–34	1.16 (0.80-1.67)	0.84 (0.46-1.52)	1.20 (0.85-1.69)		**0.56 (0.39-0.80)**	0.90 (0.52-1.58)	**0.59 (0.43-0.81)**	
35–44	1.09 (0.74-1.60)	0.63 (0.34-1.15)	1.37 (0.95-1.98)		0.73 (0.49-1.08)	0.88 (0.52-1.49)	0.94 (0.66-1.36)	
45–54	0.87 (0.61-1.24)	0.80 (0.47-1.36)	1.04 (0.77-1.40)		**0.68 (0.48-0.96)**	0.76 (0.43-1.35)	0.80 (0.60-1.07)	
55+	1				1			
**RACE/ETHNICITY**								
Hispanic	**2.46 (1.40-4.32)**	1.67 (0.79-3.53)	**1.74 (1.03-2.93)**	1	**2.63 (1.53-4.53)**	1.54 (0.73-3.25)	**1.83 (1.12-3.00)**	1
White, non-Hispanic	1				1			
Black, non-Hispanic	0.99 (0.62-1.60)	1.24 (0.69-2.23)	0.74 (0.49-1.10)		1.52 (0.91-2.54)	1.37 (0.74-2.55)	1.14 (0.74-1.76)	

	Table 2a<br>aOR Presence of Smokefree Rules—CIGARETTES (Complete vs Partial/None) among Multi- and Single-Unit Housing Residents				Table 2b<br>aOR Presence of Vapefree Rules—E-CIGARETTES (Complete vs Partial/None) among Multi- and Single-Unit Housing Residents			
**Variable**	**Unweighted N=39,624<br>Weighted N=218,409,352<br>aOR (95% CI)**				**Unweighted N=39,444<br>Weighted N=217,452,139<br>aOR (95% CI)**			
	**MUH Complete Rule**	**MUH Partial/No Rule**	**SUH Complete Rule**	**SUH Partial/No Rule (ref)**	**MUH Complete Rule**	**MUH Partial/No Rule**	**SUH Complete Rule**	**SUH Partial/No Rule (ref)**
Other, non-Hispanic	1.27(0.77-2.10)	1.13 (0.53-2.42)	0.87 (0.53-1.43)		**1.76 (1.02-3.02)**	1.47 (0.71-3.05)	1.22 (0.72-2.06)	
**U.S. CENSUS REGION**								
Northeast	**2.12 (1.35-3.32)**	**2.50 (1.37-4.55)**	1.18 (0.82-1.71)		**2.06 (1.31-3.25)**	**2.73 (1.51-4.93)**	1.16 (0.79-1.70)	
Midwest	1				1			
South	1.02 (0.70-1.50)	0.87 (0.50-1.50)	1.33 (0.98-1.80)	1	0.86 (0.60-1.25)	0.87 (0.49-1.52)	1.11 (0.84-1.46)	1
West	**1.86 (1.19-2.90)**	1.42 (0.73-2.74)	**1.78 (1.23-2.59)**		1.42 (0.94-2.13)	1.19 (0.65-2.17)	1.33 (0.96-1.85)	
**METROPOLITAN STATUS**								
Metropolitan	**3.41 (2.24-5.17)**	**3.25 (1.64-6.41)**	1.14 (0.85-1.53)	1	**3.29 (2.11-5.15)**	**3.24 (1.67-6.26)**	1.11 (0.81-1.52)	1
Non-metropolitan	1				1			
**ANNUAL HOUSEHOLD INCOME**								
<$20,000	0.99 (0.61-1.62)	1.89 (0.90-3.97)	**0.56 (0.38-0.84)**		0.96 (0.61-1.53)	1.54 (0.79-3.00)	**0.54 (0.37-0.79)**	
$20,000–$39,999	1.02 (0.65-1.59)	1.76 (0.85-3.66)	**0.66 (0.47-0.94)**	1	1.11 (0.74-1.66)	1.35 (0.73-2.52)	**0.71 (0.51-0.97)**	1
$40,000–$74,999	1.04 (0.73-1.49)	1.30 (0.62-2.76)	**0.73 (0.54-0.99)**		1.05 (0.74-1.50)	1.05 (0.57-1.92)	**0.72 (0.53-0.98)**	
$75,000+	1				1			

	Table 2a<br>aOR Presence of Smokefree Rules—CIGARETTES (Complete vs Partial/None) among Multi- and Single-Unit Housing Residents				Table 2b<br>aOR Presence of Vapefree Rules—E-CIGARETTES (Complete vs Partial/None) among Multi- and Single-Unit Housing Residents			
**Variable**	**Unweighted N=39,624<br>Weighted N=218,409,352<br>aOR (95% CI)**				**Unweighted N=39,444<br>Weighted N=217,452,139<br>aOR (95% CI)**			
	**MUH Complete Rule**	**MUH Partial/No Rule**	**SUH Complete Rule**	**SUH Partial/No Rule (ref)**	**MUH Complete Rule**	**MUH Partial/No Rule**	**SUH Complete Rule**	**SUH Partial/No Rule (ref)**
**EDUCATIONAL ATTAINMENT**								
Less than high school	**0.25 (0.15-0.41)**	**0.28 (0.14-0.54)**	**0.51 (0.35-0.74)**	1	**0.36 (0.22-0.61)**	**0.23 (0.12-0.48)**	0.73 (0.48-1.11)	1
High school	**0.33 (0.23-0.50)**	**0.35 (0.20-0.63)**	**0.59 (0.43-0.82)**		**0.41 (0.28-0.61)**	**0.37 (0.22-0.62)**	0.73 (0.53-1.01)	
Some college, no degree	**0.41 (0.27-0.93)**	**0.49 (0.26-0.93)**	**0.71 (0.51-0.99)**		**0.46 (0.30-0.85)**	**0.50 (0.30-0.85)**	0.78 (0.57-1.08)	
Associate degree	**0.55 (0.35-0.87)**	0.68 (0.36-1.31)	0.78 (0.53-1.15)		**0.57 (0.37-0.88)**	0.69 (0.37-1.27)	0.81 (0.56-1.19)	
College degree	1				1			
**EMPLOYMENT STATUS**								
Working/Not working*	1			1	1			1
Unemployed-on layoff/Unemployed-looking for work	1.19 (0.51-2.79)	1.04 (0.34-3.19)	1.12 (0.50-2.47)		1.18 (0.54-2.59)	0.97 (0.36-2.61)	1.09 (0.55-2.17)	
Not in labor force	0.73 (0.53-1.02)	0.93 (0.59-1.45)	0.79 (0.59-1.06)		0.94 (0.68-1.31)	0.76 (0.49-1.19)	0.99 (0.74-1.33)	
**HOUSING TENURE**								
Public	**63.25 (18.48-216.48)**	**55.45 (13.62-225.85)**	1.66 (0.51-5.40)	1	**60.17 (16.50-219.40)**	**58.88 (14.30-242.43)**	1.56 (0.43-5.58)	1
Private owned	1				1			

	Table 2a<br>aOR Presence of Smokefree Rules—CIGARETTES (Complete vs Partial/None) among Multi- and Single-Unit Housing Residents				Table 2b<br>aOR Presence of Vapefree Rules—E-CIGARETTES (Complete vs Partial/None) among Multi- and Single-Unit Housing Residents			
**Variable**	**Unweighted N=39,624<br>Weighted N=218,409,352<br>aOR (95% CI)**				**Unweighted N=39,444<br>Weighted N=217,452,139<br>aOR (95% CI)**			
	**MUH Complete Rule**	**MUH Partial/No Rule**	**SUH Complete Rule**	**SUH Partial/No Rule (ref)**	**MUH Complete Rule**	**MUH Partial/No Rule**	**SUH Complete Rule**	**SUH Partial/No Rule (ref)**
Private rented‡	**8.35 (5.83-11.94)**	**8.33 (4.82-14.42)**	0.74 (0.53-1.03)		**8.64 (6.30-11.85)**	**9.92 (5.67-17.37)**	0.78 (0.58-1.05)	
**SMOKING STATUS**								
Never	1				1			
Current	**0.08 (0.06-0.10)**	0.83 (0.53-1.30)	**0.09 (0.07-0.12)**	1	**0.08 (0.06-0.11)**	0.73 (0.47-1.13)	**0.10 (0.08-0.13)**	1
Former	**0.67 (0.50-0.91)**	1.01 (0.59-1.72)	**0.70 (0.53-0.91)**		**0.37 (0.28-0.49)**	1.09 (0.70-1.70)	**0.40 (0.31-0.51)**	

Notes:

1. Estimates are based on weighted adjusted polytomous/multinomial logistic regression model.
2. Results of the multinomial model are reported separately in Table 2a and Table 2b; they compare housing/rule categories to SUH Partial/No (smokefree or vapefree) rule as the referent group.
3. Multi-unit housing is defined as two or more housing units that share a wall or ceiling. Single-unit housing refers to independent housing units such as single-family homes, mobile homes, and other housing units that do not share a wall or ceiling.
4. Complete rule indicates that respondents reported no one is allowed to smoke/vape anywhere inside their home. Partial/no rule indicates that respondents reported that smoking/vaping is allowed in some places or at some times inside their home or reported that smoking/vaping is permitted anywhere inside their home, respectively.

* Working refers to respondents who are employed-at work. Not working includes those who are with job, not at work; employed-absent; unemployed-on layoff; and unemployed-looking for work.

‡Rent includes no cash rent or rent without payment.

Supplemental Table S3. Home Smoking and Vaping Rules and Support for Indoor Multi-Unit Housing Smoking Bans in Multi- and Single-Unit Housing by Housing Tenure, 2019

	MULTI-UNIT HOUSING						SINGLE-UNIT HOUSING					
**Variable**	**Population (N=57,524,958)**	**Total (n=9,258)**	**Public (n=873)**	**Private owned (n=2,520)**	**Private rented* (n=5,865)**	***p*-value**	**Population (N=156,215,832)**	**Total (n=29,472)**	**Public (n=185)**	**Private owned (n=24,720)**	**Private rented (n=4,567)**	***p*-value**
**HOME SMOKING RULES**						<.0001						<.0001
No one is allowed to smoke anywhere inside your home	51,535,954	8,287	728 (83.8)	2,312 (91.7)	5,247 (89.6)		141,829,710	26,562	158 (86.6)	22,502 (91.8)	3,902 (85.6)	
Smoking is allowed in some places or at some times inside your home	2,918,554	446	49 (5.4)	106 (4.6)	291 (5.2)		7,749,238	1,459	13 (3.3)	1,104 (4.2)	342 (8.3)	
Smoking is permitted anywhere inside your home	3,070,449	525	96 (10.8)	102 (3.8)	327 (5.2)		7,055,548	1,451	14 (10.2)	1,114 (4.1)	323 (6.1)	
**HOME VAPING RULES**						<.0001						<.0001
No one is allowed to vape anywhere inside your home	49,951,211	8,121	747 (85.1)	2,287 (90.2)	5,087 (85.8)		140,332,126	26,465	164 (87.5)	22,444 (91.0)	3,857 (84.4)	

	MULTI-UNIT HOUSING						SINGLE-UNIT HOUSING					
**Variable**	**Population (N=57,524,958)**	**Total (n=9,258)**	**Public (n=873)**	**Private owned (n=2,520)**	**Private rented* (n=5,865)**	***p*-value**	**Population (N=156,215,832)**	**Total (n=29,472)**	**Public (n=185)**	**Private owned (n=24,720)**	**Private rented (n=4,567)**	***p*-value**
Vaping is allowed in some places or at some times inside your home	3,322,913	479	34 (3.9)	102 (4.7)	343 (6.4)		7,050,190	1,302	8 (2.4)	977 (3.8)	317 (7.6)	
Vaping is permitted anywhere inside your home	4,250,833	658	92 (11.0)	131 (5.1)	435 (7.8)		8,833,516	1,705	13 (10.1)	1,299 (5.1)	393 (7.9)	
**PERCEPTIONS OF SMOKING IN BUILDINGS WITH MULTIPLE APARTMENTS OR LIVING AREAS**						<.0001						<.0001
Allowed inside all apartments or living areas	5,545,074	889	110 (11.6)	263 (10.6)	516 (9.0)		12,990,745	2,457	13 (6.6)	2,022 (8.1)	422 (8.9)	
Allowed inside some apartments	12,007,970	1,933	163 (18.4)	528 (20.3)	1,242 (21.4)		36,911,846	6,982	43 (19.5)	5,851 (23.6)	1,088 (23.2)	
Not allowed at all inside apartments	39,971,913	6,436	600 (70.0)	1,729 (69.1)	4,107 (69.6)		106,731,905	20,033	129 (73.9)	16,847 (68.2)	3,057 (67.9)	

Notes:

1. All values are number (weighted percentage) unless otherwise indicated.
2. P-values for all chi-squared tests were statistically significant at $p<.0001$.
3. Multi-unit housing is defined as two or more housing units that share a wall or ceiling. Single-unit housing refers to independent housing units such as single-family homes, mobile homes, and other housing units that do not share a wall or ceiling.

*Rent includes no cash rent or rent without payment.

Supplemental Table 4. Home Smoking and Vaping Rules and Support for Indoor Multi-Unit Housing Smoking Bans in Multi- and Single-Unit Housing by Smoking Status, 2019

	MULTI-UNIT HOUSING						SINGLE-UNIT HOUSING					
**Variable**	**Population (N=57,452,142)**	**Total (n=9,246)**	**Never smoking (n=6,421)**	**Current smoking (n=1,069)**	**Former smoking (n=1,756)**	***p*-value**	**Population (N=155,877,872)**	**Total (n=29,419)**	**Never smoking (n=19,746)**	**Current smoking (n=3,252)**	**Former smoking (n=6,421)**	***p*-value**
**HOME SMOKING RULES**						<.0001						<.0001
No one is allowed to smoke anywhere inside your home	51,470,788	8,276	6,063 (94.0)	609 (56.4)	1,604 (90.8)		141,270,257	26,515	18,790 (95.1)	1,838 (58.1)	5,887 (91.9)	
Smoking is allowed in some places or at some times inside your home	2,910,905	445	200 (3.5)	171 (16.9)	74 (4.4)		7,632,942	1,455	570 (3.0)	602 (18.7)	283 (4.3)	
Smoking is permitted anywhere inside your home	3,070,449	525	158 (2.4)	289 (26.7)	78 (4.8)		6,974,673	1,449	386 (1.9)	812 (23.2)	251 (3.8)	
**HOME VAPING RULES**						<.0001						<.0001
No one is allowed to vape anywhere inside your home	49,886,046	8,110	5,962 (91.7)	651 (58.8)	1,497 (82.7)		140,021,707	26,418	18,742 (94.7)	1,971 (60.3)	5,705 (88.0)	

	MULTI-UNIT HOUSING						SINGLE-UNIT HOUSING					
**Variable**	**Population (N=57,452,142)**	**Total (n=9,246)**	**Never smoking (n=6,421)**	**Current smoking (n=1,069)**	**Former smoking (n=1,756)**	***p*-value**	**Population (N=155,877,872)**	**Total (n=29,419)**	**Never smoking (n=19,746)**	**Current smoking (n=3,252)**	**Former smoking (n=6,421)**	***p*-value**
Vaping is allowed in some places or at some times inside your home	3,315,263	478	249 (4.5)	120 (12.1)	109 (7.6)		7,042,778	1,300	562 (2.9)	411 (13.6)	327 (5.5)	
Vaping is permitted anywhere inside your home	4,250,833	658	210 (3.8)	298 (29.1)	150 (9.7)		8,813,388	1,701	442 (2.4)	870 (26.1)	389 (6.6)	
**PERCEPTIONS OF SMOKING IN BUILDINGS WITH MULTIPLE APARTMENTS OR LIVING AREAS**						<.0001						<.0001
Allowed inside all apartments or living areas	5,545,074	889	432 (7.0)	286 (28.2)	171 (9.7)		12,866,537	2,448	1,148 (5.9)	605 (18.8)	695 (11.3)	
Allowed inside some apartments	11,998,479	1,931	1,187 (19.1)	334 (30.1)	410 (23.1)		36,655,616	6,968	4,107 (20.8)	1,141 (35.3)	1,720 (27.2)	
Not allowed at all inside apartments	39,908,589	6,426	4,802 (73.9)	449 (41.7)	1,175 (67.2)		106,355,720	20,003	14,491 (73.3)	1,506 (45.9)	4,006 (61.6)	

Notes:

1. All values are number (weighted percentage) unless otherwise indicated.
2. P-values for all chi-squared tests were statistically significant at $p<.0001$.
3. Multi-unit housing is defined as two or more housing units that share a wall or ceiling. Single-unit housing refers to independent housing units such as single family homes, mobile homes, and other housing units that do not share a wall or ceiling.
